# Supplementary material for: High-frequency Contactless Sensor for the Detection of Heparin-Induced Thrombocytopenia Antibodies via Platelet Aggregation
Source: Int J Mol Sci. 2022 Nov 19;23(22):14395. doi: 10.3390/ijms232214395 (PMC9694871; doi:10.3390/ijms232214395)
Supplement: Supplementary file 1 [file ijms-23-14395-s001.zip › ijms-1964456-supplementary.pdf]

Supplementary Information

High-frequency Contactless Sensor for the Detection of Heparin-Induced Thrombocytopenia Antibodies *via* Platelet Aggregation

Nida Zaman Khan,[1, 2] Daniel Martin,[1] Uwe Pliquett,[1, 2] Yahor Zaikou,[1] Nacke Thomas,[1] Doris Heinrich,[1, 2] J. Michael Köhler,[2] and Thi-Huong Nguyen[1, 2]*

[1]*Institute for Bioprocessing and Analytical Measurement Techniques (iba), 37308 Heiligenstadt, Germany*

[2]*Institute for Chemistry and Biotechnology, Faculty of Mathematics and Natural Sciences, Technische Universität Ilmenau, 98694 Ilmenau, Germany*

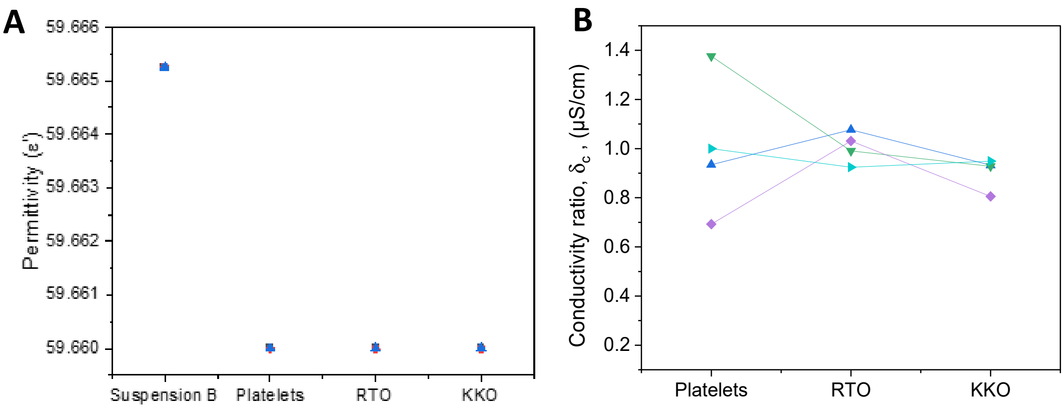

Figure S1. (A) Platelet samples show lower permittivity compared to suspension buffer. No different permittivity was observed among platelet alone, and platelet with the addition of RTO or KKO antibodies. (**B**) No significant change in the ratio of conductivity δ_c was seen due to the effect of platelet concentration, platelet age, and bubble-effect.
